# Hypertension control rate in India: systematic review and meta-analysis of population-level non-interventional studies, 2001–2022

**DOI:** 10.1016/j.lansea.2022.100113

**Published:** 2022-11-23

**Authors:** Shaffi Fazaludeen Koya, Zarin Pilakkadavath, Praseeda Chandran, Tom Wilson, Serin Kuriakose, Suni K. Akbar, Althaf Ali

**Affiliations:** a Boston University School of Public Health, Boston, USA; b Boston University School of Medicine, Boston, USA; c Department of Community Medicine, Government Medical College, Manjeri, India; d Department of Community Medicine, Kannur Medical College, Anjarakandy, India; e National Centre for Disease Control, New Delhi, India; f KIMS Al-Shifa Specialty Hospital, Perinthalmanna, India; g Department of Community Medicine, Government Medical College, Thiruvananthapuram, India

**Keywords:** Hypertension, Control, Blood pressure, Risk factors, Social determinants, Prevalence, Meta-analysis

## Abstract

**Background:**

Hypertension is a significant contributor to mortality in India. Achieving better hypertension control rate at the population level is critical in reducing cardiovascular morbidity and mortality.

**Methods:**

Hypertension control rate was defined as the proportion of patients with their blood pressure under control (systolic blood pressure <140 mmHg and diastolic blood pressure <90 mmHg). We conducted a systematic review and meta-analysis of community-based, non-interventional studies published after 2001 that reported hypertension control rates. We searched PubMed, Embase, and Web of Science databases, and grey literature, and extracted data using a common framework, and summarized the study characteristics. We conducted random-effects meta-analysis using untransformed hypertension control rates and reported the overall summary estimates and subgroup estimates of control rates as percentages and 95% confidence intervals. We also conducted mixed-effects meta-regression with sex, region, and study period as covariates. The risk of bias was assessed, and level of evidence was summarized using SIGN-50 methodology. The protocol was pre-registered with PROSPERO, CRD42021267973.

**Findings:**

The systematic review included 51 studies (n = 338,313 hypertensive patients). 21 studies (41%) reported poorer control rates among males than females, and six studies (12%) reported poorer control rates among rural patients. The pooled hypertension control rate in India during 2001–2020 was 17.5% (95% CI: 14.3%–20.6%)—with significant increase over the years, reaching 22.5% (CI: 16.9–28.0%) in 2016–2020. Sub-group analysis showed significantly better control rates in the South and West regions, and significantly poorer control rates among males. Very few studies reported data on social determinants or lifestyle risk factors.

**Interpretation:**

Less than one-fourth of hypertensive patients in India had their blood pressure under control during 2016–2020. Although the control rate has improved compared to previous years, substantial differences exist across regions. Very few studies have examined the lifestyle risk factors and social determinants relevant to hypertension control in India. The country needs to develop and evaluate sustainable, community-based strategies and programs to improve hypertension control rates.

**Funding:**

Not applicable.

**Research in context**

**Evidence before this study**

Hypertension is a major modifiable risk factor for cardiovascular diseases and stroke. Two-thirds of the world's hypertensives resides in low- and middle-income countries. Although easily diagnosable and amenable to management, hypertension control remains a public health challenge. This may be attributed to the fact that while 50% of the population affected with hypertension are aware of their high blood pressure, only around half of them get treated. Moreover, only 25% achieve blood pressure control among those who get treated. Most recent systematic review on hypertension control in India published in 2014 showed significant urban-rural differences.

**Added value of this study**

We conducted a systematic review on hypertension control in India covering three databases and two sources of grey literature. We focused on community-based non-interventional studies which provide a realistic picture of control rates at the community level. We examined the changes in control rates over the years, which, to the best of our knowledge, was never done before. We also examined the availability of literature on risk factors of hypertension control including social determinants of health.

**Implication of all available evidence**

Hypertension control is strongly influenced by health system factors, lifestyle risk factors, and social determinants. The paucity of data in the Indian literature on these critical factors—as our review showed—should prompt serious efforts toward developing nationally representative studies to capture the extent and the key determinants of hypertension control in India. The overall low rates of control, and significant differences in control rates between regions should trigger more research and actions aimed at improving the current national program.

## Introduction

Hypertension is an important modifiable risk factor for cardiovascular diseases (CVD), making it one of the significant contributors of premature death and morbidity.^1^^,^^2^ Worldwide, the age-adjusted prevalence of hypertension has plateaued, but the absolute number has doubled due to an increasing trend in low- and middle-income countries (LMICs) between 2000 and 2010.^3^ Globally, only 21% of hypertensive patients had their blood pressure under control in 2021.^1^ Hypertension is the most important risk factor for death and disability in India.^4^^,^^5^ The 2019–2020 National Family Health Survey (NFHS-5) reported a hypertension prevalence of 24% in men and 21% among women, an increase from 19% and 17% respectively from the previous round (2015–16).^6^

Recognizing the increasing burden of non-communicable diseases (NCDs), India formally launched its National Program for Prevention and Control of Cancer, Diabetes, Cardiovascular Diseases and Stroke (NPCDCS) in 2010, and subsequently expanded the program across country by March 2016.^7^ However, close to 80% of NCD patients in India who seek medical care from the private sector^8^ are not covered by the program. These patients are not actively monitored for hypertension control including but not limited to medication adherence.^8^ Less than 8% of hypertensive patients had their blood pressure under control as per 2015-16 NFHS data.^6^ Responding to the poor control rates and to improve the access to treatment services, a new program—the India Hypertension Control Initiative (IHCI) was launched in 2017 as a multi-partner initiative of the Government of India's Ministry of Health & Family Welfare, Indian Council of Medical Research (ICMR), WHO Country Office for India, and Resolve to Save Lives.^9^

Although recently published data from the IHCI have reported improvement in control rates among patients receiving care through health centers, the population-level control rates in the project areas remained abysmally low.^10^ There have been no published systematic reviews or meta-analysis on hypertension control in India in the recent years. (see *research in context*) The last available systematic review and meta-analysis on hypertension control in India published in 2014^11^ did not explore the changes in control rates over the years.

This paper addresses these gaps by providing an updated systematic review of available literature and a meta-analysis by examining population-based studies in the last 20 years. Specifically, we aimed to systematically describe the characteristics of the published literature and to document the changes in hypertension control rates over the years at the population level. The current review, therefore, answers the following questions:

1. What does the literature show about the population-level hypertension control rate in India?
2. What are the population-level sex-specific and region-specific estimates of hypertension control rates in India?
3. Whether population-level hypertension control rates in India have improved over the years?

## Methods

This review is guided by the Preferred Reporting Items for Systematic Reviews and Meta-Analyses (PRISMA) statement.^12^ For the data extraction, we used a “descriptive-analytical” method proposed by Hilary Arksey & Lisa O'Malley which involves applying a common framework to all the papers included to collect standard information on key issues and themes.^13^ Institutional review board approval was not required for this study since no patient identifiers were included in the study or analysis. The review protocol was registered with the PROSPERO database (CRD42021267973).

### Search strategy

We first searched PubMed, Web of Science, and Embase on 31 July 2021 for all peer-reviewed papers published between 1 January 2001 and 31 December 2020. We used the same search strings later to update the search with studies from 1 January 2021 through 22 September 2022. The search strategy (see Supplement S1) used a combination of MeSH and non-MeSH terms for ‘hypertension’ and ‘control’ in PubMed and equivalent terms in Web of Science and Embase. Additionally, we conducted a grey literature search on 25 September 2022 in google scholar and in Shodhganga which is an Indian Electronic Theses and Dissertation repository (https://shodhganga.inflibnet.ac.in/). We examined the first 40 pages (400 articles) in google scholar (search terms: “India” AND “hypertension” AND “control”) which did not give any new publication other than what we already found in PubMed, Web of Science, and Embase. The search in Shodhganga (search: “hypertension,∖ control” without any filter) showed 306 thesis/dissertations. The title screening showed that none of them met the inclusion criteria, as the population studies were either interventions or trials, while the rest of the studies were laboratory-based.

### Study eligibility

We included all original population-level non-interventional studies published since 1 January 2001 in this review. We excluded studies on secondary hypertension, interventional studies, qualitative studies, hospital-based studies, commentaries, and reviews. Studies which used convenience sampling and studies which did not provide the total number of hypertensive patients were excluded.

### Data charting and extraction

One author [SFK] downloaded all the records from the databases, de-duplicated them using Zotero, and uploaded them to Rayyan online collaborative systematic review platform.^14^ Four authors (SFK, ZP, PC, and TW) screened all the titles and abstracts using Rayyan. This was followed by a detailed reading of the screened-in papers by at least two authors following the inclusion criteria. In the final stage, the following data were extracted from the included papers independently by two authors using a spreadsheet: authors, published year, study/data collection year, state, geographical area covered (rural/urban), sample size (sex-disaggregated), definitions of hypertension and control, total hypertension cases and percentage (disaggregated across sex), control rates (total number and percentage, disaggregated numbers and percentages across sex), reported difference in control rates across rural/urban, education levels, income status (rich/poor), antihypertension treatment status, and details of blood pressure measurement. Disagreements between reviewers were sorted out through discussions and pending discrepancies were resolved by the lead reviewer. Whenever there was ambiguity, the lead reviewer was consulted, and a decision was taken on consensus.

All papers included in the systematic review are listed in Supplement (S2—A) along with the key variables extracted. A map (StataCorp. 2021) showing the distribution of studies across Indian states (after excluding studies covering multiple states) is given in the Supplement (S2–B). The details of the blood pressure measurement are included in Supplement (S2–C). These include number of measurements, device details, wait time, sitting arrangement, and interval between measurements.

### Definitions

Hypertension was defined as having either a systolic blood pressure (SBP) at least 140 mmHg, and/or a diastolic blood pressure (DBP) at least 90 mmHg, and/or history of medication(s) to lower BP. Controlled blood pressure was defined as systolic blood pressure (SBP) less than 140 mmHg and diastolic blood pressure (DBP) less than 90 mmHg as per the Joint National Committee 7 (JNC 7) classification.^15^ We calculated hypertension control rate as the proportion of patients with their blood pressure under control as defined above. We included all hypertensive patients in the denominator to calculate the rate, irrespective of whether they were newly diagnosed at the time of study or their medication history.

### Study quality assessment and level of evidence

We used a previously validated tool by Hoy et al.^16^ which has been used to estimate bias in prevalence studies including Global Burden of Diseases estimates^17^ and in recent systematic reviews on hypertension control.^18^ To briefly summarize, the tool measures internal and external validity, and rates the bias—measurement, selection, and analysis bias— as either high or low risk using a ten-item questionnaire, and then make an overall assessment of the risk of bias rated as either low, moderate, or high risk. Two authors [PC and SFK] rated each of the papers using this tool and any disagreement was resolved through discussion. Additionally, the studies were assessed for the level of evidence (“high quality”, “low risk of bias”, and “high risk of bias”) as per the SIGN-50 methodology following Winter CF et al.^19^ (Supplement S3).

### Statistical analysis

First, we described the study characteristics using numbers and proportions to summarize the available literature. Second, we summarized the difference in hypertension prevalence and control rates based on sex, geography (rural/urban), education levels, income status, and by antihypertension medication status as reported by the authors. We used the p-values or 95% confidence intervals to decide whether the differences between groups were significant. Lastly, we conducted the meta-analysis and meta-regression using the untransformed (raw) hypertension control rates as the summary effect size statistic which was found to be normally distributed in Q–Q plot. We report the control rates for the whole period and for blocks of five years.

### Test of heterogeneity

Since the studies came from different regions of the country and have different population characteristics, we anticipated heterogeneity and therefore decided to use the random effect model *a priori*. We used multiple methods to examine heterogeneity in our data. First, we created a forest plot to visually inspect the data. Second, we looked at the total amount of systematic differences in effects across studies calculated as the between-study variance (heterogeneity, measured as τ^2^ (tau-squared)) and standard deviation (τ). We used the DerSimonian-Laird estimator^20^ to calculate the heterogeneity variance (τ^2^) and the Jackson method^21^ to calculate its 95% Confidence Intervals (CI) with Knapp-Hartung adjustments.^22^ Third, we estimated the I^2^ statistic (with 95% CIs)^23^ which is the ratio of observed heterogeneity (between-study variance) and the total observed variance (sum of within-study variance due to sampling error and between-study variance). Lastly, we conducted a formal χ^2^ test with a Cochran's Q statistic, to test if all studies share the common effect size.^24^

### Meta-analysis

We used the random effects model to calculate the summary effect size, i.e., the weighted average of the observed control rates in each study. The inverse of the total variance of the study was used to weigh each study. When the initial random effects model with Hartung-Knapp adjustment revealed high levels of heterogeneity (number of studies included = 47) (Supplement S4), we used a diagnostic Baujat plot to identify outliers and influential studies causing heterogeneity. The diagnostic Baujat plot and test of residuals (Supplement S5A—B) showed two studies with studentized residuals (z-values) greater than three. Further, we performed a set of leave-one-out diagnostic tests (Supplement S6) to calculate the summary values of hypertension control rates by excluding one study each at a time from the analysis. Based on these tests, we removed two outliers from the final pooled analysis, thus the main meta-analysis includes 45 studies.

### Subgroup analysis

We conducted separate subgroup analysis across sex, region, and years to understand the heterogeneity. For the subgroup analysis across years, we compared control rates across studies conducted in blocks of five years (2001–2005, 2006–2010, 2011–2015, 2016–2020). For the subgroup analysis across sex (male, female), we conducted meta-analysis of 194,439 hypertensive patients (145,883 females) pooled from 29 studies with sex-segregated data. For the subgroup analysis across regions (North, East, South, West), we excluded studies with data from multiple regions and analyzed 38,685 hypertensive patients from 35 studies (18 studies from south, 9 from north, 5 from east, and 3 from west). We used forest plot and tables to summarize results of the subgroup analysis.

### Meta-regression

Lastly, we undertook a *post hoc* mixed-effects meta-regression^25^^,^^26^ to examine the role of region, period of study, and sex as mediators. We used the following model equation: $\hat{\theta_{k}}=\theta +\beta x_{k}+\varepsilon_{k}+\zeta_{k}\;,$ where $\hat{\theta_{k}}$ is the observed effect-size (hypertension control rate) with *k* studies, θ is the intercept, *β* is the regression coefficient for the variable *x*, $\varepsilon_{k}$ is the sampling error, and $\zeta_{k}$ is the error arising due to heterogeneity. We built the multiple regression model after excluding studies with data from multiple regions.

### Sensitivity analysis

We conducted two sensitivity analyses. The first analysis was done by avoiding four studies that had data on elderly population only. In the second analysis, we included only those studies with a low risk of bias.

### Publication bias

With prevalence as the outcome measure, we did not expect publication bias arising from study design related significance level,^27^ and therefore we did not examine publication bias in this review.

All the statistical tests were two-sided, and the p-value was fixed at 0.05. We conducted all analyses using R software version 4.1.1 (R Core Team, 2020); we used the “tidyverse” package for systematic review summary tables, the “metafor” and “meta” packages for the meta-analysis, and the “robvis” package for risk of bias visualization.^28^, ^29^, ^30^

## Results

### Summary of study characteristics

**Fig. 1:**
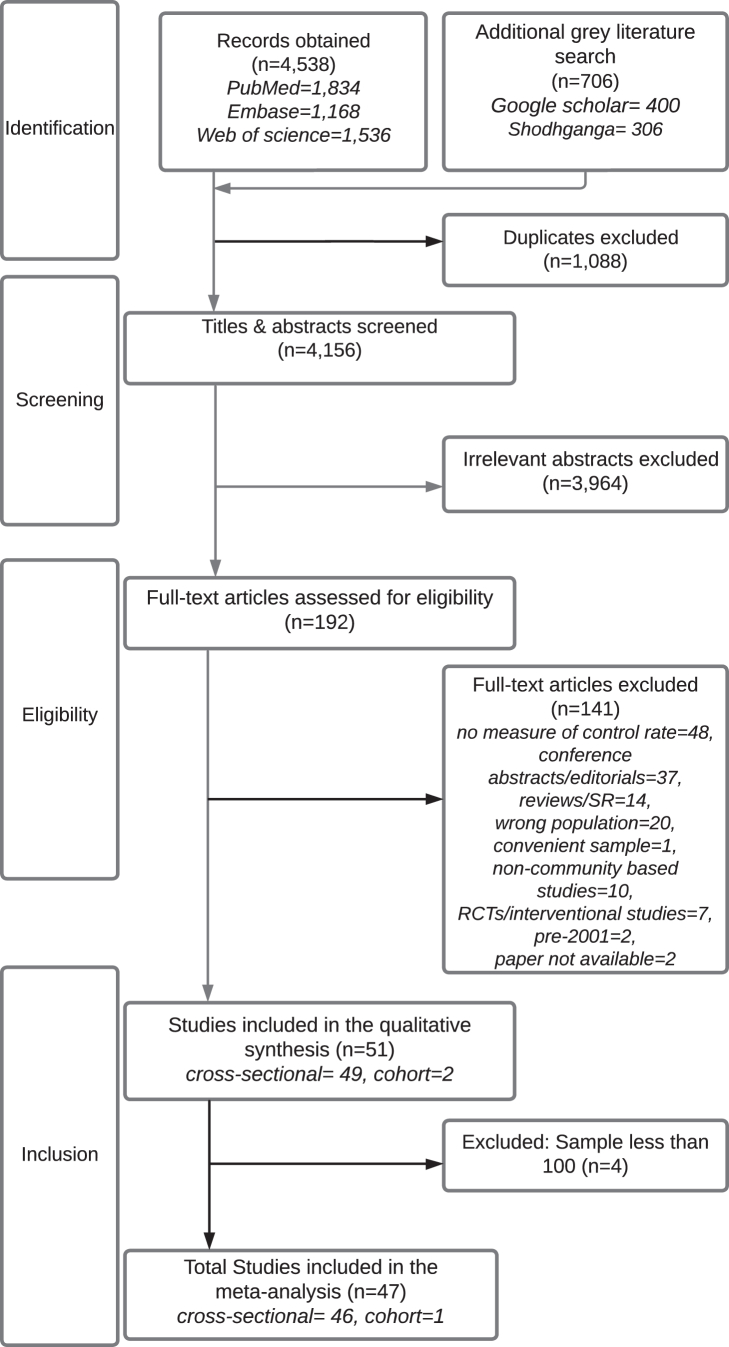
PRISMA Flow diagram showing the study selection for the systematic review and meta-analysis. Note: PRISMA: Preferred Reporting Items for Systematic Reviews and Meta-Analyses.

Fig. 1 shows the screening and review stages. We excluded 3964 irrelevant articles after title and abstract screening. After a detailed reading of each of the remaining 192 papers by at least two authors, we excluded 141 articles due to the following reasons: missing data, wrong article type, wrong population, wrong study period, intervention studies, or full paper not available. The final systematic review included 51 articles. Of these, four articles that had less than 100 hypertensive patients were excluded from the meta-analysis due to unstable results from an extremely large ratio of largest to smallest sampling variance.

The review included 49 cross-sectional and two cohort studies covering 1.39 million population (73% females) including 338,313 hypertensive patients (average = 6634 patients/study). Thirty nine studies had state-specific data covering 15 states and territories (Supplement (S2–B)). The mean hypertension prevalence across studies was 24.2%, and 46.8% (n = 158,252) of them were aware of their high blood pressure. The overall characteristics of the included studies are given in Table 1. 20 studies (39%) used systematic random sampling, 16 (31%) used cluster sampling and 10 (20%) used simple random sampling. 12% of studies (n = 6) had data from all the states and 9.8% of studies (n = 5) had data from multiple states. 20% (n = 10) of studies had data from more than one region of the country. 69% of studies had sex-disaggregated data on hypertension control rates. 33% of studies (n = 17) had data from the pre-NPCDCS years (2001–2010), while the remaining 67% (n = 34) had data after the launch of the NPCDCS.

**Table 1:** Summary characteristics of studies included in the systematic review.

Characteristics	N = 51^a^
**Sex**	
Both males and females	48 (94.1%)
Females only	2 (3.9%)
Males only	1 (2.0%)
**Age groups**	
15 years and above	3 (5.9%)
18 years and above	41 (80.0%)
65 years and above only	7 (14%)
**Region**	
East	6 (12%)
North	12 (24%)
South	20 (39%)
West	3 (5.9%)
More than one region	10 (20%)
**Area**	
Both rural and urban areas	20 (39%)
Rural areas only	19 (37%)
Urban areas only	12 (24%)
**Study (data collection) period**	
Pre-NCD program period (2001–2010)	17 (33%)
2001–2005	3 (5.9%)
2006–2010	14 (27%)
NCD program period (2011—)	34 (67%)
2011–2015	18 (35%)
2016–2020	16 (31%)
**Period of publication**	
2001–2010	8 (16%)
2011–2022	43 (84%)
**Study design**	
Cohort	2 (3.9%)
Cross-sectional	49 (96%)
**Sampling method**	
Census	3 (5.9%)
Cluster sampling	16 (31%)
Simple random sampling	10 (20%)
Systematic random sampling	20 (39%)
Not reported	2 (3.9%)

a Number (%).

21 studies (41%) reported poorer control rates among males compared to females and three studies (5.9%) reported poorer control rates among females compared to males. Six studies (12%) reported poorer control rates among rural patients compared to urban patients, four studies reported no difference in control rates between rural and urban patients, while two studies (3.9%) reported poorer control among urban patients. Three studies each (5.9%) reported poorer controls among low income/wealth group patients and less educated patients. 16% (n = 8) of studies reported no difference in control rate across the educational level. 78% (n = 40) of studies did not have data on differences in educational level.

**Fig. 2:**
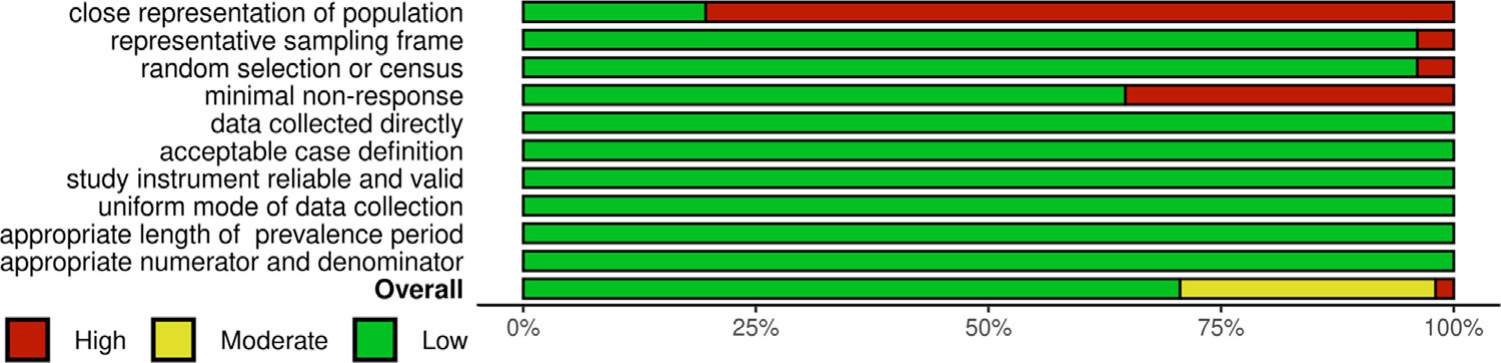
Summary of authors' judgement on risk of bias. Note: The risk of bias was assessed using a previously validated tool (Hoy et al.) Summary plot created using robvis R package.

There were 36 studies with low risk of bias, 14 with moderate risk of bias, and one with high risk of bias (Fig. 2 and Supplement S7). Two studies received a SIGN-50 score of LE2 indicating a high risk of bias while the remaining 49 studies had a score of LE2++ indicating high-quality evidence (Supplement S7).

### Hypertension control rate

**Fig. 3:**
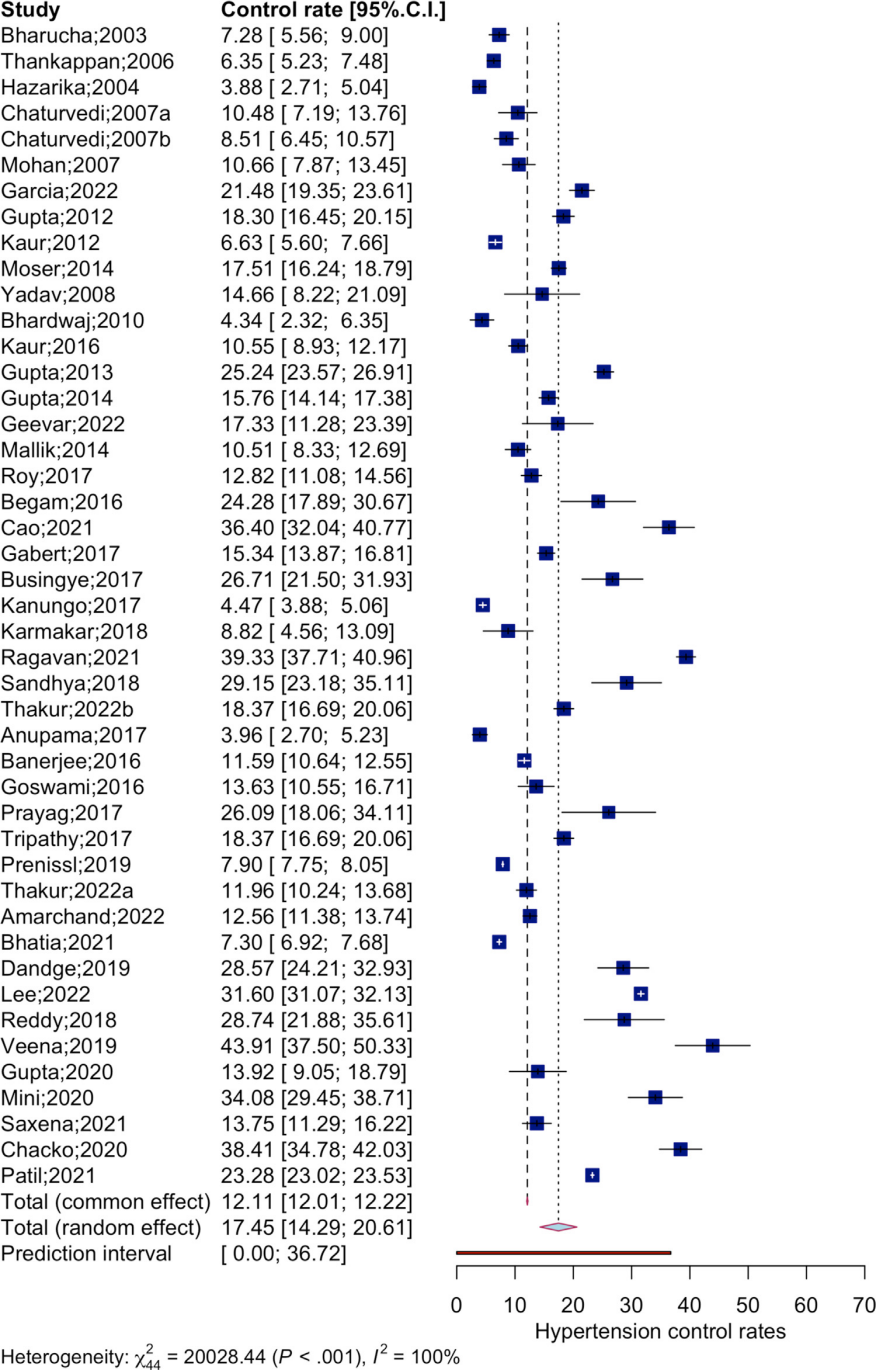
Forest plot of meta-analysis of hypertension control rates in population-level non-interventional studies in India, 2001–2022. *Note:* Values expressed in percentage.

The pooled analysis is summarized as a forest plot (Fig. 3). The random effects model analyzed data of 53,908 patients with blood pressure under control among the total 336,835 hypertensive patients from 45 studies. The pooled hypertension control rate in India during 2001–2020 was 17.5% (95% CI: 14.3%–20.6%). The model provided a wide prediction interval, 0.0%–36.7% reflecting higher level of heterogeneity (τ^2^ = 0.0089 [0.0048; 0.0181]; τ = 0.0945 [0.0696; 0.1347], I^2^ = 99.8%; H = 21.34,Q _(*df* = 44)_ = 20028.44, p-value< 0.001).

### Subgroup analysis

The results from the subgroup analysis are summarized as forest plots (Supplement Fig. S8). We found that the control rates significantly increased over the years (Supplement S8—A): from 5.8% (CI: 0–17.2%) in 2001-’05; to 13.7% (CI: 7.9%–19.4%) in 2006–2010; 18.5% (CI: 13.6%–23.3%) in 2011–2015; and to 22.5% (CI: 16.9–28.0%) in 2016–2020), p = 0.028. There was significant difference in control rates between males and females—13.7% (CI: 9.9–17.5%) in males and 20.4% (CI: 16.7–24.0%) in females, p = 0.012 (Supplement S8—B). Further, the control rates improved significantly for both sex during the period, although the magnitude of improvement was higher among females [6.7% in 2001–2005 (CI: 1.0–12.5%) to 25.4% in 2016–20 (CI: 17.3–33.5%), p < 0.0001] compared to males [4.6% in 2001–2005 (CI: 2.1–7.1%) to 14.8% in 2016–20 (CI: 9.3–20.3%), p < 0.0001]. Control rates were significantly different across regions (p < 0.0001). The southern region (23.5%, CI:17.3–29.8%) reported the highest control rate and the eastern region (7.8%, CI:3.5–12.2%) reported the lowest (Supplement S8—C).

Results of the meta-regression are summarized in Supplement Table S9. The model with sex, study period, and regions explains 61.6% of heterogeneity. There is a significant moderator effect of sex, region, and study period— males had significantly poorer control rates, the southern region had significantly higher control rates, and the control rates during 2011–2015 and 2015–2020 were significantly higher. We report these results with caution as meta-regression does not necessarily demonstrate a causal relationship but indicates an association.

### Sensitivity analysis

After we excluded studies with data only on the elderly (Supplement S10—A), the pooled control rate was 17.6% (CI: 14.0%–21.1%) which was not different from the control rate in the main model. Similarly, the control rate from the model with “low risk of bias” studies (Supplement S10—B) did not differ from the control rate in the main model— 15.3% (CI:12.2%–18.5%)

## Discussion

In this paper, we have summarized the literature on population-level hypertension control rates in India over the last 20 years and provided updated summary estimates of control rates. To our understanding this is the first meta-analysis that examined the changes in control rates over years. There are four key findings from our review.

First, only 22.5% of hypertensive patients in India have their blood pressure under control during the most recent period in our analysis— 2016 to 2020. The previously published meta-analysis of community-level hypertension control in India with data from 1950 to 2013 showed a control rate of 10.7% for rural India and 20.2% for urban India.^9^ In comparison, the control rate during the 20 years in our study was 17.5%. This poor rate is a matter of concern considering that only 50% of patients in 15–49-year age group in India knew their hypertension status as per the NFHS-4 data (2015–16).^31^ However, the control rate in India is similar to those reported from other countries. The US CDC reported a comparable control rate of 26.1% in the US during 2013–18.^32^ A review from China recently reported that the control rate ranged between 4.2 and 30.1%,^33^ while a recent meta-analysis from Ghana reported a control rate of 6.0% (CI: 3.0%–10.0%).^34^ A recent cross-sectional study of 1.1 million adults across 44 LMICs including India showed that the hypertension control rate was 10.3%.^35^ A systematic review and meta-analysis from Nepal showed a hypertension control rate of 38% among treated hypertensives with marginal improvement over years,^36^ and this is similar to our rate among treated patients. The most recent data from Pakistan shows that half of diagnosed hypertensive patients are treated and 12.5% have their blood pressure under control.^37^

Second, the hypertension control rates have significantly improved in the last two decades. The rates increased nearly four times from 5.8% in 2001–2005 to 22.5% in 2016–2020. However, considering the increasing prevalence of hypertension especially among the poor^38^ and young adults,^39^ and very high proportion of patients unaware of their high blood pressure (53% in our review), India's national program needs to revisit its goals and strategies to improve the control rates in India. The program needs to consider the learnings from recent interventions, especially from the ongoing IHCI project in several states and the Mumbai Hypertension Project. A recent study^40^ which analyzed the initial cohort from four Indian states under IHCI, reported significant improvement in blood pressure control rates (59.8% in follow up versus 26.3% at baseline). However, the improvement in control rates was observed among patients receiving care from the health centers but this was not translated to overall improvement in the community level control rates. Similarly, the Mumbai Hypertension Project^41^ also found that the effect of the project was limited directly to the project beneficiaries and not at the population level. This evidence should prompt the redesigning of national program into a more sustainable, expandable, and decentralized model of hypertension care continuum involving community health workers including the Accredited Social Health Activists (ASHA) and incorporating the services of private sector which provides a significant proportion of hypertension control services in India. Interrupted supply of medicines, inadequate health education, and poor health literacy can have a synergistic effect leading to incomplete treatment or non-compliance. The results from IHCI and Mumbai showed the importance of community level follow-up mechanisms to ensure continuity of care including regular blood pressure examinations. This necessitates remotely supervised delegation of hypertension management by community health workers, considering shortage of doctors in rural areas.

Third, significant regional differences exist in the hypertension control, albeit we found very few studies in the West and the North India compared to the South. South India showed better control rates, and southern states of Kerala and Tamil Nadu states reported the highest rates of control. The difference in health systems’ capacity to detect and treat hypertension varies across the country as much as the level of awareness about the disease, its prevention, and control. Treatment adherence and access to medicine are key determinants of blood pressure control. Veena et al. reported that among those with controlled hypertension, 23.7% subjects monitored blood pressure 2–4 times a year while 67.30% never monitored their blood pressure.^42^ The one study in our review which examined adherence to medications^43^ showed significant association between regular medication and control rate. We found two studies^39^^,^^44^ that compared control status based on treatment status, while no study was found to examine the access to antihypertensive medicines. A recent review had shown that poor availability of generic medicines in public and private sector and high costs are major barriers to antihypertensive control including in India.^45^ Another study reported that around 70% of the estimated proportion of adults with hypertension did not receive antihypertensive drugs in 2018.^46^ Besides, the high out-of-pocket expenditure and lack of insurance coverage for out-patient services and drugs reduces the access to anti-hypertensive medication, resulting in uncontrolled hypertension.^9^

Fourth, very few studies examined the association of lifestyle and other risk factors with poor control rates. Among them, Tripathy et al.^47^ reported that uncontrolled hypertension was more frequent among obese patients, patients with sedentary lifestyle, and diabetic patients. Thankappan et al.^48^ also reported poor blood pressure control among diabetic and obese patients. Diet and smoking,^43^ higher percent body fat^49^ and good family support to self-care were shown to predict the blood pressure control.^43^

Lastly, very few studies in the review had data on key social determinants of hypertension control, like education, income, wealth, and caste. 11 (21.6%) studies in the review had data on education, while seven (13.7%) studies had data on income/wealth/socioeconomic status. A 13%-point gap in hypertension control rate between the rich and the poor and a clear disadvantage for scheduled castes, tribes and backward communities were reported in the recent Longitudinal Ageing Study in India.^50^

Our study has a few limitations. We searched three databases for peer-reviewed studies and two sources for grey literature. Therefore, we could have missed studies available in other sources. Although we used a validated tool to examine the study bias, the raters’ subjective judgment might have resulted in an under or overestimation of study quality. Considering the substantial heterogeneity, the pooled control rate cannot be interpreted as a national average. However, with a fairly high quality of evidence used in our review, we provide a reasonably reliable information on changes in control rate over the years and differences across regions— which have policy and program implications.

### Conclusion

India needs better data at the community level to understand the problem of hypertension control, especially in the North and West regions. The country may plan studies to collect nationally representative data at regular intervals which can help us better understand the differences in control rates across regions and subpopulations. These studies should examine relevant health-system factors, lifestyle factors, and social and structural determinants including income, wealth, employment, cultural barriers, and caste that influence blood pressure control levels. These data can inform and guide policies and programs to specifically address the key determinants of uncontrolled hypertension in India.

## Contributors

S.F.K: Conceptualization, Methodology, Formal analysis, Investigation, Resources, Writing – original draft, Supervision. Z.P: Formal analysis, Investigation, Writing – review & editing. P.C: Formal analysis, Investigation, Writing – review & editing. T.W: Investigation, Writing – review & editing. S.K: Investigation, Writing – review & editing. SKA: Writing – review & editing. AA: Resources, Formal analysis, Investigation, Writing – review & editing.

S.F.K, and Z.P had full access to all the data used in the study. SFK was responsible for the decision to submit the manuscript.

## Data sharing statement

The data used for analysis are available within the paper and supplementary files.

## Declaration of interests

S.F.K's salary from 10.13039/100007161Boston University is partly supported by the 3-D Commission project under the 10.13039/100000877Rockefeller Foundation grant (2019 HTH 024).

The authors declare no conflict of interests.
