# Supplementary material for: Hypertension control rate in India: systematic review and meta-analysis of population-level non-interventional studies, 2001–2022
Source: Lancet Reg Health Southeast Asia. 2022 Nov 23;9:100113. doi: 10.1016/j.lansea.2022.100113 (PMC10305851; doi:10.1016/j.lansea.2022.100113)
Supplement: Supplementary File S1 [file mmc1.pdf]

Hypertension control rate in India: Systematic review and meta-analysis of population level non-interventional studies, 2001-2022

Shaffi Fazaludeen Koya, DrPH, Zarin Pilakkadavath, DNB, Praseeda Chandran MD et al.

Supplement files

S1 Search strategy

PubMed

("Hypertension"[Mesh] OR "hypertension"[Text Word] OR "blood pressure"[Text Word] OR "raised blood pressure"[Text Word] OR "elevated blood pressure"[Text Word] OR "systolic blood pressure"[Text Word] OR "diastolic blood pressure"[Text Word] OR "SBP"[Text Word] OR "DBP"[Text Word] OR "isolated systolic blood pressure"[Text Word] OR "high BP"[Text Word] OR "BP"[Text Word] OR "raised BP"[Text Word] OR "elevated BP"[Text Word]) AND ("India"[Text Word] OR "Indian"[Text Word]) AND "control"[Text Word] AND 2001/01/01:2022/09/20[Date - Publication]

Web of science

((TI=('hypertension'/exp OR hypertension OR 'blood pressure' OR 'raised blood pressure' OR 'elevated blood pressure' OR 'systolic blood pressure' OR 'diastolic blood pressure' OR 'sbp' OR 'dbp' OR 'isolated systolic blood pressure' OR 'high bp' OR 'bp' OR 'raised bp' OR 'elevated bp')) AND TI=('indian' OR 'india')) AND TI=('control') AND ('indian' OR 'india') AND ('control')) AND [2001-2022]/py

Embase

('hypertension':ab,ti OR hypertension:ab,ti OR 'blood pressure':ab,ti OR 'raised blood pressure':ab,ti OR 'elevated blood pressure':ab,ti OR 'systolic blood pressure':ab,ti OR 'diastolic blood pressure':ab,ti OR 'sbp':ab,ti OR 'dbp':ab,ti OR 'isolated systolic blood pressure':ab,ti OR 'high bp':ab,ti OR 'bp':ab,ti OR 'raised bp':ab,ti OR 'elevated bp':ab,ti) AND ('indian':ab,ti OR india:ab,ti) AND 'control':ab,ti AND (2001:py OR 2002:py OR 2003:py OR 2004:py OR 2005:py OR 2006:py OR 2007:py OR 2008:py OR 2009:py OR 2010:py OR 2011:py OR 2012:py OR 2013:py OR 2014:py OR 2015:py OR 2016:py OR 2017:py OR 2018:py OR 2019:py OR 2020:py OR 2021:py OR 2022:py) AND [embase]/lim NOT ([embase]/lim AND [medline]/lim)

S2—A Studies included in the systematic review (n= 51): Key variables extracted

Sl. No	Author; Year	Study Year	Study Design	State	Region	Area	Sex	Sampling Design	Age Groups	HTN Prev: %	Known Prev: %	HTN Prev: Sex	HTN Prev: Area	Control Rate: %	Male Control Rate: %	Female Control Rate: %
1	Amarchand;2022	2017	C/S	AI	MR	U/R	M/F	SyRS	18-69	28.5	27.8	M>F	U>R	12.6	10.3	15.3
2	Anupama;2017	2015	C/S	KT	S	R	M/F	Cl/S	adults	30.4	27.4	F=M	UA/NA	4	UA/NA	UA/NA
3	Banerjee;2016	2015	C/S	WB	E	U	M/F	SRS	adults	42	54.4	M>F	UA/NA	11.6	9.7	13.1
4	Begam;2016	2013	C/S	KL	S	R	M	SyRS	adults	45	48	UA/NA	UA/NA	24.3	24.3	UA/NA
5	Bhardwaj;2010	2009	C/S	HP	N	R	M/F	Cl/S	adults	35.9	21.4	M>F	UA/NA	4.3	UA/NA	UA/NA
6	Bharucha;2003	2001	C/S	MH	W	U	M/F	Cl/S	adults	36.4	52.3	UA/NA	UA/NA	7.3	5.6	8.5
7	Bhatia;2021	2017	C/S	AI	MR	U/R	M/F	SyRS	45+	27.4	73	UA/NA	UA/NA	7.3	UA/NA	UA/NA
8	Busingye;2017	2014	C/S	AP	S	R	M/F	SyRS	adults	##	42.6	M>F	UA/NA	26.7	25.7	28
9	Cao;2021	2013	C/S	KL	S	R	M/F	Cl/S	30+	27.3	54.4	M>F	UA/NA	36.4	26.9	54.7
10	Chacko;2020	2019	C/S	KL	S	U/R	M/F	Cl/S	adults	##	NA	UA/NA	UA/NA	38.4	41	36.7
11	Chaturvedi;2007a	2006	C/S	DL	N	U/R	M/F	Cl/S	adults	27.5	53.3	F=M	UA/NA	10.5	6.7	14
12	Chaturvedi;2007b	2006	C/S	DL	N	U/R	M/F	Cl/S	elderly	63.8	54	F=M	UA/NA	8.5	8.9	8
13	Dandge;2019	2017	C/S	TL	S	R	M/F	SRS	adults	23.6	61	M>F	UA/NA	28.6	UA/NA	UA/NA
14	Gabert;2017	2013	C/S	**	MR	U/R	M/F	SyRS	15+	32.2	37.4	F>M	UA/NA	15.3	UA/NA	UA/NA
15	Garc;2022	2007	C/S	AI	MR	U/R	M/F	NM	60+	41.4	46.1	UA/NA	UA/NA	21.5	UA/NA	UA/NA
16	Geevar;2022	2011	C/S	KL	S	U/R	M/F	SRS	20-39	12.3	34.7	M>F	U>R	17.3	2.5	1.9
17	Godara;2021	2019	C/S	RJ	N	R	M/F	Cl/S	30+	22	27.3	M>F	UA/NA	9.1	6.4	15.8
18	Goswami;2016	2015	C/S	DL	N	U	M/F	Cl/S	elderly	67.2	41.3	F>M	UA/NA	13.6	7.2	18
19	Gupta;2012	2007	C/S	AI	MR	U/R	F	SyRS	adults	39.2	43.5	UA/NA	U>R	18.3	UA/NA	18.3
20	Gupta;2013	2010	C/S	**	MR	U	M/F	Cl/S	adults	42.8	57	M>F	UA/NA	25.2	24.4	26.3
21	Gupta;2014	2010	C/S	**	MR	U	M/F	Cl/S	adults	31.3	55.3	F>M	UA/NA	15.8	13.7	18.1
22	Gupta;2020	2018	C/S	HR	W	R	M/F	SRS	elderly	50.3	58.8	F=M	UA/NA	13.9	12.2	15
23	Hazarika;2004	2003	C/S	AS	E	R	M/F	SRS	adults	33.3	21.6	F=M	UA/NA	3.9	3.6	4.1
24	Kanungo;2017	2014	C/S	WB	E	U/R	M/F	SyRS	adults	26	16.1	M>F	U>R	4.5	UA/NA	UA/NA
25	Kapoor;2021	2020	C/S	**	N	U/R	M/F	NM	50+	##	##	UA/NA	UA/NA	53.8	49.9	56.8
26	Karmakar;2018	2014	C/S	WB	E	R	M/F	SyRS	adults	26.1	48.2	F>M	UA/NA	8.8	10.4	7.8
27	Kaur;2012	2007	C/S	TN	S	R	M/F	SRS	adults	21.5	25.1	F=M	UA/NA	6.6	6.1	7.1
28	Kaur;2016	2009	C	TN	S	R	M/F	SRS	adults	22.9	24.5	UA/NA	UA/NA	10.5	UA/NA	UA/NA
29	Kharat;2021	2019	C/S	PO	S	U	M/F	SyRS	adults	##	##	UA/NA	UA/NA	57.4	51	62
30	Kusuma;2013	2007	C/S	DL	N	U	M/F	Cl/S	adults	18.3	41	M>F	UA/NA	6	0	13.5
31	Lee;2022	2017	C/S	**	MR	U/R	M/F	SyRS	45+	45.9	58.2	nd	R=U	31.6	12.8	18

Sl. No	Author; Year	Study Year	Study Design	State	Region	Area	Sex	Sampling Design	Age Groups	HTN Prev: %	Known Prev: %	HTN Prev: Sex	HTN Prev: Area	Control Rate: %	Male Control Rate: %	Female Control Rate: %
32	Mallik;2014	2011	C/S	WB	E	R	M/F	Cl/S	adults	41.9	40.1	UA/NA	UA/NA	10.5	UA/NA	UA/NA
33	Mini;2020	2018	C/S	KL	S	U/R	M/F	SyRS	adults	14.6	61.9	M>F	UA/NA	34.1	22	39.1
34	Misra;2014	2011	C/S	AS	E	R	M/F	Cl/S	adults	26	23.5	F=M	UA/NA	2.4	UA/NA	UA/NA
35	Mohan;2007	2006	C/S	TN	S	U	M/F	SyRS	adults	20	32.8	M>F	UA/NA	10.7	UA/NA	UA/NA
36	Moser;2014	2007	C/S	KL	S	U/R	M/F	Cl/S	adults	32	41.1	F>M	U>R	17.5	14.2	19.8
37	Patil;2021	2019	C/S	AI	MR	U/R	M/F	SRS	adults	29.4	43.8	UA/NA	UA/NA	23.3	UA/NA	UA/NA
38	Prayag;2017	2015	C/S	KT	S	U	M/F	SyRS	adults	38.3	53	F>M	UA/NA	26.1	UA/NA	UA/NA
39	Prenissl;2019	2016	C/S	AI	MR	U/R	M/F	Cl/S	15+	17.8	44.7	M>F	U>R	7.9	5.3	10.9
40	Ragavan;2021	2014	C/S	**	S	R	M/F	SyRS	adults	29.6	64.2	F>M	UA/NA	39.3	UA/NA	UA/NA
41	Reddy;2018	2017	C/S	TL	S	U	M/F	SRS	elderly	83.5	80.8	F>M	UA/NA	28.7	UA/NA	UA/NA
42	Roy;2017	2012	C/S	DL	N	U/R	M/F	Cl/S	adults	35.7	38.7	M>F	U>R	12.8	7	18.6
43	Sandhya;2018	2014	C/S	KL	S	R	F	SyRS	adults	32.1	67.7	UA/NA	UA/NA	29.1	UA/NA	29.1
44	Sathish;2012	2006	C	KL	S	R	M/F	SyRS	15+	21.4	##	M>F	UA/NA	2.9	UA/NA	UA/NA
45	Saxena;2021	2018	C/S	UK	N	R	M/F	SyRS	adults	39	43.5	M>F	UA/NA	13.8	7.6	20.6
46	Thakur;2022	2014	C/S	PB	N	U/R	M/F	SyRS	18-69	40.3	48.3	M>F	R>U	18.4	9.1	25.3
47	Thakur;2022	2016	C/S	HR	N	U/R	M/F	SyRS	18-69	26.2	33.3	M>F	U>R	12	9.8	14.4
48	Thankappan;2006	2001	C/S	KL	S	R	M/F	Cl/S	adults	36.7	24.4	F>M	UA/NA	6.4	4.6	7.6
49	Tripathy;2017	2015	C/S	PB	W	U/R	M/F	SyRS	adults	40.1	30.1	M>F	R=U	18.4	9.1	29.1
50	Veena;2019	2017	C/S	PO	S	U	M/F	Cl/S	adults	##	##	UA/NA	UA/NA	43.9	UA/NA	UA/NA
51	Yadav;2008	2007	C/S	DL	N	U	M/F	SRS	elderly	39.5	51.7	M>F	UA/NA	14.7	UA/NA	UA/NA

*Note: HTN: Hypertension, Prev: Prevalence, C/S: Cross-sectional, C: Cohort, S: South, N: North, E: East, W: West, MR- Multiple regions, R: Rural, U: Urban, U/R: Urban and rural, M: Male, F: Female, M/F: Males and Females, Cl/S: Cluster sampling, SRS: Simple random sampling, SyRS: Systematic random sampling, UA: Data unavailable, NA: Not applicable, F↓: Females have poorer control rate, M ↓: Males have poorer control rate, nd: No difference, ##: Only hypertensive patients included in the study, Elderly: 65 and above, AP: Andhra Pradesh, AS: Assam, DL: Delhi, TL: Telangana, WB: West Bengal, HP: Himachal Pradesh, UK: Uttarakhand, MH: Maharashtra, TN: Tamil Nadu, KT: Karnataka, HR: Haryana, PB: Punjab, PO: Pondicherry, AI: All India, **: Multiple states*

S2—B **Map showing the distribution of studies on hypertension control rate, 2001-2022**

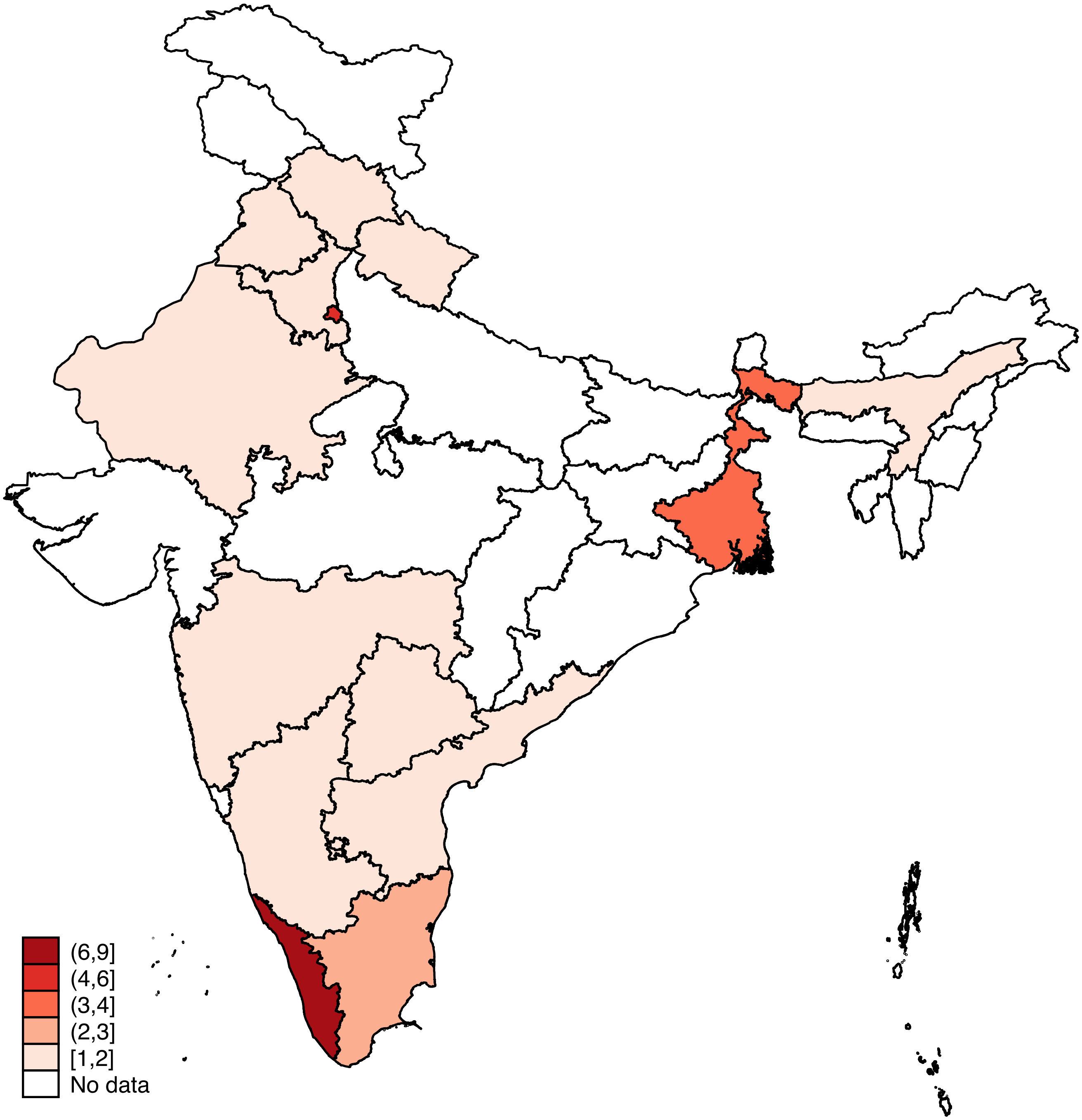

S2—C Blood pressure measurement details from studies included in the systematic review (n= 51)

Author; Year	Number of Measurements	Device Details	Wait Time	Sitting Arrangement	Interval Between Measurement
Amarchand;2022	3	Automatic BP machines (OMRON HEM–7120, Omron Corporation, Kyoto, Japan)	not mentioned	sitting	5 minutes
Anupama;2017	2	mercury sphygmomanometers (Diamond, Pune, India)	not mentioned	sitting	5 minutes
Banerjee;2016	2	aneroid sphygmomanometers	not mentioned	not mentioned	2 minutes
Begam;2016	3	automatic Omron BP apparatus (OMRON-4, Omron Corporation, Kyoto, Japan)	not mentioned	not mentioned	3 minutes
Bhardwaj;2010	2	mercury sphygmomanometer	not mentioned	not mentioned	5 minutes
Bharucha;2003	2	Hawksley random zero Sphygmomanometer	not mentioned	sitting	5 minutes
Bhatia;2021	2	not mentioned	not mentioned	not mentioned	not mentioned
Busingye;2017	3	Digital automatic BP monitor (OMRON HEM-907, OMRON Healthcare Company, Kyoto, Japan)	15 minutes	sitting	3 minutes
Cao;2021	3	Omron automatic BP monitor (model IA2)	not mentioned	not mentioned	3 minutes
Chacko;2020	3	digital sphygmomanometer (OMRON HEM-7121)	not mentioned	sitting	3 minutes
Chaturvedi;2007a	3	mercury sphygmomanometer	not mentioned	sitting	2 minutes

Author; Year	Number of Measurements	Device Details	Wait Time	Sitting Arrangement	Interval Between Measurement
Chaturvedi;2007b	3	mercury sphygmomanometer	not mentioned	sitting	2 minutes
Dandge;2019	3	Rossmax (model MJ701f) automated sphygmomanometer	not mentioned	sitting	1 minute
Gabert;2017	not mentioned	a digital sphygmomanometer	not mentioned	not mentioned	not mentioned
Garc;2022	3	not mentioned	not mentioned	not mentioned	not mentioned
Geevar;2022	3	electronic BP apparatus (model 1A2, Omron Corporation, Shimogyo-ku, Kyoto, Japan)	15 minutes	sitting	3 minutes
Godara;2021	3	not mentioned	not mentioned	not mentioned	not mentioned
Goswami;2016	2	digital sphygmomanometer (Omron automatic blood pressure monitor, model HEM-7130)	not mentioned	sitting	5 minutes
Gupta;2012	3	digital sphygmomanometer supplied centrally (Omron model SDX, Omron Inc., USA)	5 minutes	sitting	2 minutes
Gupta;2013	3	Omron SDX instruments (Omron Healthcare, Lake Forest, IL)	not mentioned	sitting	5 minutes
Gupta;2014	3	standardized instrument	9 minutes	sitting	not mentioned
Gupta;2020	2	digital blood pressure machine (Omron automatic blood pressure monitor, model: HEM-7120)	30 minutes	sitting	5 minutes

Author; Year	Number of Measurements	Device Details	Wait Time	Sitting Arrangement	Interval Between Measurement
Hazarika;2004	3	mercury column sphygmomanometer	15 minutes	sitting	10 to 15 minutes
Kanungo;2017	2	Rossmax – AW150 Blood Pressure Monitor (Rossmax International Ltd., Berneck, Switzerland)	not mentioned	not mentioned	10 minutes
Kapoor;2021	3	aneroid sphygmomanometer.	15 minutes	sitting	not mentioned
Karmakar;2018	not mentioned	not mentioned	not mentioned	not mentioned	not mentioned
Kaur;2012	2	electronic automatic blood pressure apparatus (Omron MX3	5 minutes	sitting	5 minutes
Kaur;2016	2	electronic automatic BP apparatus (Omron MX3)	10 minutes	sitting	not mentioned
Kharat;2021	3	not described	not mentioned	not mentioned	not mentioned
Kusuma;2013	3	mercury column sphygmomanometer	not mentioned	sitting	not mentioned
Lee;2022	3	Omron automatic BP monitor	not mentioned	not mentioned	not mentioned
Mallik;2014	3	mercury sphygmomanometer	5 minutes	sitting	5 minutes
Mini;2020	3	digital Omron blood pressure apparatus (OMRON-4, Omron Corporation, Kyoto, Japan)	11 minutes	sitting	not mentioned
Misra;2014	3	Omron digital automatic blood pressure monitor three times (OMRON -4, Omron Corporation, Kyoto, Japan)	not mentioned	sitting	not mentioned

Author; Year	Number of Measurements	Device Details	Wait Time	Sitting Arrangement	Interval Between Measurement
Mohan;2007	not mentioned	not mentioned	not mentioned	not mentioned	not mentioned
Moser;2014	3	Boso Medistar Wrist Blood Pressure Monitor Model S	not mentioned	sitting	1 minute
Patil;2021	3	not mentioned	not mentioned	not mentioned	not mentioned
Prayag;2017	2	mercury sphygmomanometer	10 minutes	sitting	5 minutes
Prenissl;2019	3	portable Omron BP monitor, model HEM-8712)	5 minutes	sitting	5 minutes
Ragavan;2021	3	digital automatic BP monitor (OMRON HEM-907, OMRON Healthcare Company, Kyoto, Japan)	15 minutes	sitting	3 minutes
Reddy;2018	3	electronic sphygmomanometer (OMRON HEM 7120, Omron Healthcare Co., Ltd., Japan)	30 minutes	sitting	1 minute
Roy;2017	2	automated blood pressure machine (OMRON (HEM-7080)	not mentioned	sitting	2 minutes
Sandhya;2018	not mentioned	not mentioned	not mentioned	not mentioned	not mentioned
Sathish;2012	2	OMRON digital automatic BP monitor (OMRON-4, Omron Corporation, Kyoto, Japan	12 minutes	sitting	not mentioned
Saxena;2021	2	Omron digital Blood pressure instrument	6 minutes	sitting	3 minutes

Author; Year	Number of Measurements	Device Details	Wait Time	Sitting Arrangement	Interval Between Measurement
Thakur;2022	2	calibrated electronic equipment (OMRON HEM 7120)	not mentioned	not mentioned	2 minutes
Thakur;2022	2	calibrated electronic equipment (OMRON HEM 7120)	not mentioned	not mentioned	2 minutes
Thankappan;2006	2	not mentioned	not mentioned	not mentioned	10 minutes
Tripathy;2017	3	electronic equipment (OMRON HEM 7120, Omron Corporation, Kyoto, Japan)	7 minutes	sitting	3 minutes
Veena;2019	2	OMRON HEM-7120-IN validated automated BP measuring device	not mentioned	not mentioned	not mentioned
Yadav;2008	2	mercury sphygmomanometer	8 minutes	sitting	one week

S3 Criteria used for SIGN50 score- level of evidence[1]

Score	Quality/bias	Criteria
LE2++	High quality	High-quality case–control or cohort studies with a very low risk of confounding or bias and a high probability that the relationship is causal
		High-quality cross-sectional studies with statistical analysis and very low risk of bias
LE2+	Low risk of bias	Well-conducted case–control or cohort studies with a low risk of confounding or bias and a moderate probability that the relationship is causal
		Well-conducted cross-sectional studies with statistical analysis and low risk of bias
LE2	High risk of bias	Case–control or cohort studies with a high risk of confounding or bias and a significant risk that the relationship is not causal
		Cross-sectional studies with statistical analysis and a high risk of bias
LE3	High risk of bias	Cross-sectional studies without any statistical analysis

[1]de Winter CF, Jansen AAC, Evenhuis HM. Physical conditions and challenging behaviour in people with intellectual disability: a systematic review. *Journal of Intellectual Disability Research*. 2011;55(7):675-698. doi:10.1111/j.1365-2788.2011.01390.x

S4 Forest plot of the initial pooled analysis with 47 studies.

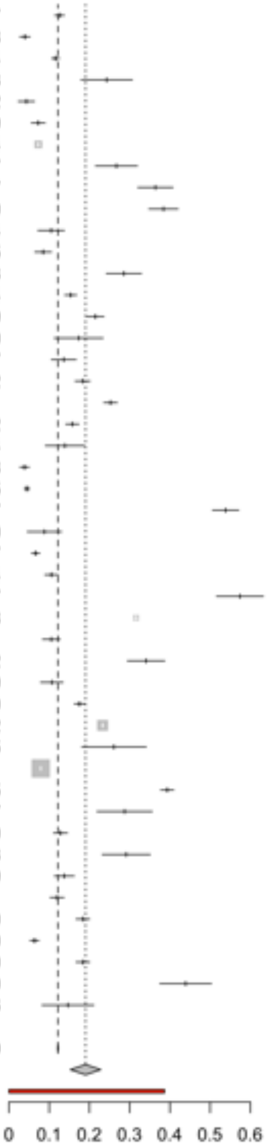

Study	Events	Total	Proportion	95%-CI	Weight (common)	Weight (random)
Amarchand;2022	379	3017	0.13	[0.11; 0.14]	0.8%	2.2%
Anupama;2017	36	908	0.04	[0.03; 0.05]	0.7%	2.2%
Banerjee;2016	499	4304	0.12	[0.11; 0.13]	1.3%	2.2%
Begam;2016	42	173	0.24	[0.18; 0.31]	0.0%	2.0%
Bhardwaj;2010	17	392	0.04	[0.02; 0.06]	0.3%	2.2%
Bharucha;2003	64	879	0.07	[0.06; 0.09]	0.4%	2.2%
Bhatia;2021	1311	17964	0.07	[0.07; 0.08]	7.9%	2.2%
Busingye;2017	74	277	0.27	[0.22; 0.32]	0.0%	2.0%
Cao;2021	170	467	0.36	[0.32; 0.41]	0.1%	2.1%
Chacko;2020	265	690	0.38	[0.35; 0.42]	0.1%	2.1%
Chaturvedi;2007a	35	334	0.10	[0.07; 0.14]	0.1%	2.1%
Chaturvedi;2007b	60	705	0.09	[0.06; 0.11]	0.3%	2.2%
Dandge;2019	118	413	0.29	[0.24; 0.33]	0.1%	2.1%
Gabert;2017	355	2314	0.15	[0.14; 0.17]	0.5%	2.2%
Garcia;2022	307	1429	0.21	[0.19; 0.24]	0.3%	2.2%
Geevar;2022	26	150	0.17	[0.11; 0.23]	0.0%	2.0%
Goswami;2016	65	477	0.14	[0.11; 0.17]	0.1%	2.1%
Gupta;2012	306	1672	0.18	[0.16; 0.20]	0.3%	2.2%
Gupta;2013	659	2611	0.25	[0.24; 0.27]	0.4%	2.2%
Gupta;2014	306	1942	0.16	[0.14; 0.17]	0.4%	2.2%
Gupta;2020	27	194	0.14	[0.09; 0.19]	0.0%	2.1%
Hazarika;2004	41	1058	0.04	[0.03; 0.05]	0.8%	2.2%
Kanungo;2017	210	4695	0.04	[0.04; 0.05]	3.3%	2.2%
Kapoor;2021	483	897	0.54	[0.51; 0.57]	0.1%	2.1%
Karmakar;2018	15	170	0.09	[0.05; 0.13]	0.1%	2.1%
Kaur;2012	149	2247	0.07	[0.06; 0.08]	1.1%	2.2%
Kaur;2016	146	1384	0.11	[0.09; 0.12]	0.4%	2.2%
Kharat;2021	159	277	0.57	[0.52; 0.63]	0.0%	2.0%
Lee;2022	9255	29288	0.32	[0.31; 0.32]	4.0%	2.2%
Mallik;2014	80	761	0.11	[0.08; 0.13]	0.2%	2.2%
Mini;2020	137	402	0.34	[0.29; 0.39]	0.1%	2.1%
Mohan;2007	50	469	0.11	[0.08; 0.13]	0.1%	2.2%
Moser;2014	599	3420	0.18	[0.16; 0.19]	0.7%	2.2%
Patil;2021	24795	106522	0.23	[0.23; 0.24]	17.8%	2.2%
Prayag;2017	30	115	0.26	[0.18; 0.34]	0.0%	1.9%
Prenissl;2019	10380	131391	0.08	[0.08; 0.08]	53.8%	2.2%
Ragavan;2021	1359	3455	0.39	[0.38; 0.41]	0.4%	2.2%
Reddy;2018	48	167	0.29	[0.22; 0.36]	0.0%	1.9%
Roy;2017	182	1420	0.13	[0.11; 0.15]	0.4%	2.2%
Sandhya;2018	65	223	0.29	[0.23; 0.35]	0.0%	2.0%
Saxena;2021	103	749	0.14	[0.11; 0.16]	0.2%	2.2%
Thakur;2022a	164	1371	0.12	[0.10; 0.14]	0.4%	2.2%
Thakur;2022b	373	2030	0.18	[0.17; 0.20]	0.4%	2.2%
Thankappan;2006	115	1810	0.06	[0.05; 0.07]	0.9%	2.2%
Tripathy;2017	373	2030	0.18	[0.17; 0.20]	0.4%	2.2%
Veena;2019	101	230	0.44	[0.37; 0.50]	0.0%	2.0%
Yadav;2008	17	116	0.15	[0.08; 0.21]	0.0%	2.0%
**Common effect model**		**338009**	**0.12**	**[0.12; 0.12]**	**100.0%**	**--**
**Random effects model**			**0.19**	**[0.15; 0.23]**	**--**	**100.0%**
**Prediction interval**				**[0.00; 0.39]**		

Heterogeneity: $I^2 = 100\%$, $\tau^2 = 0.0093$, $p = 0$

S5—A **Diagnostic Bajaut plot**

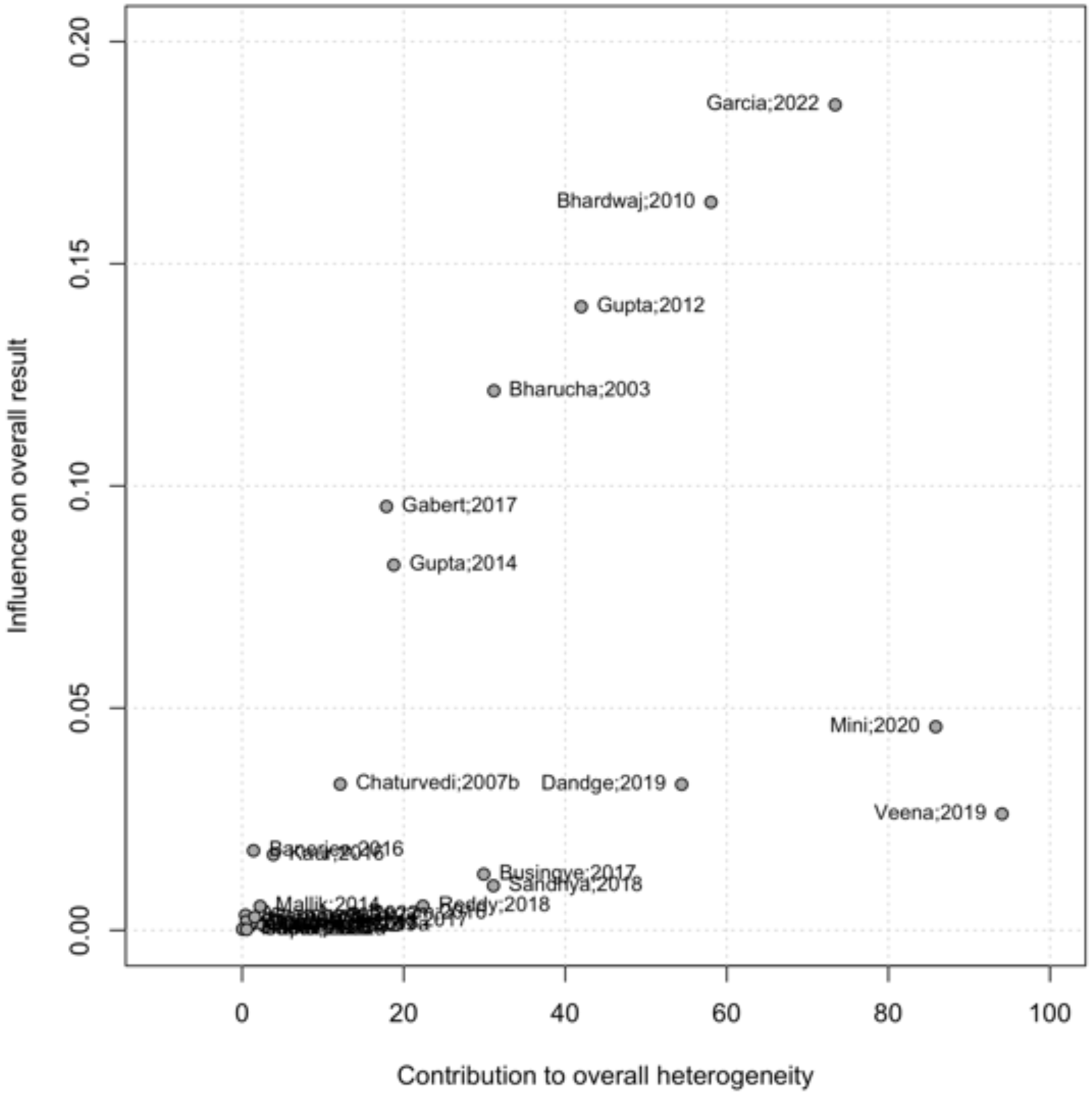

S5—B Test of residual

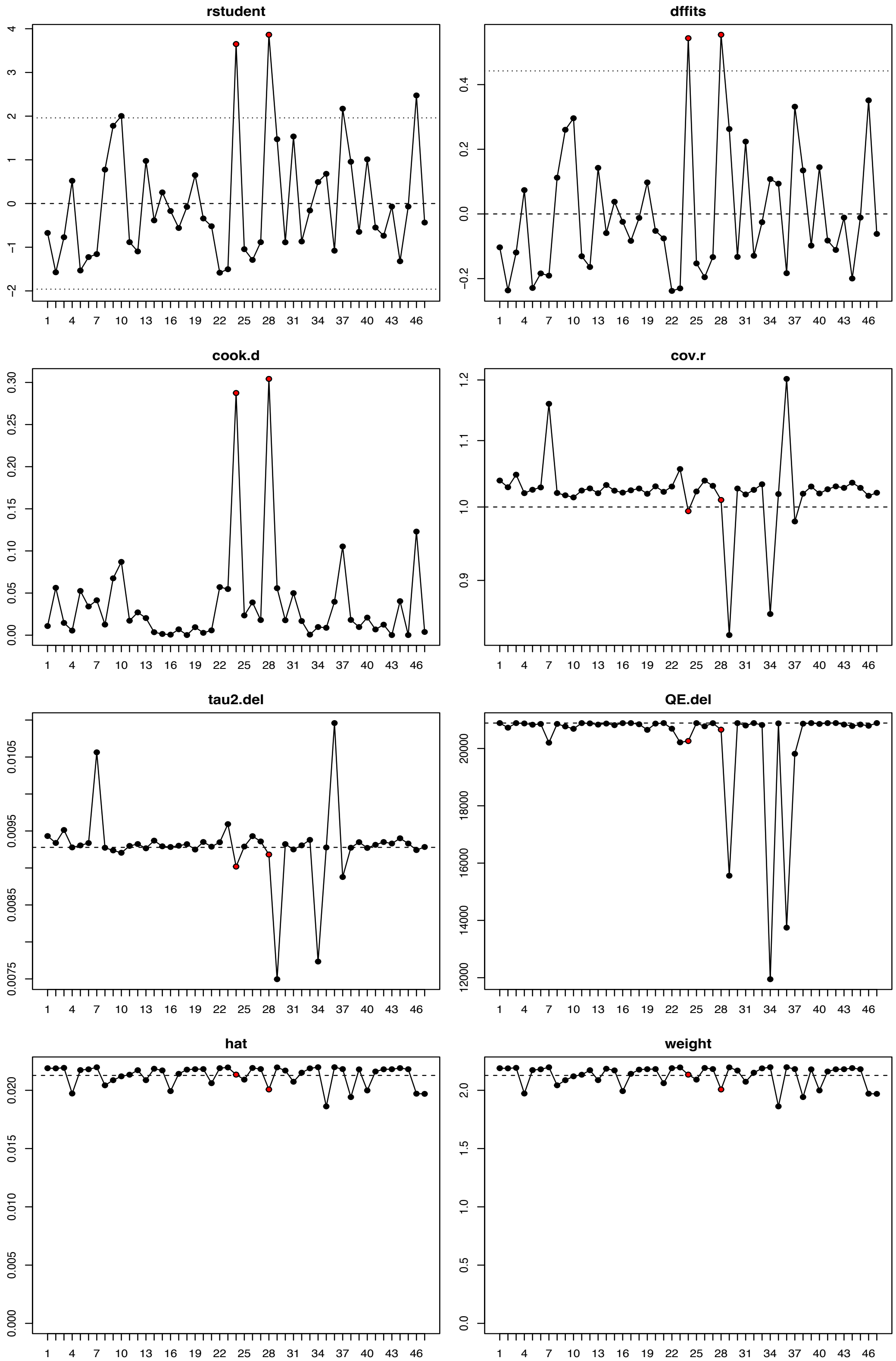

S6 Forest plot of leave-one-out analysis

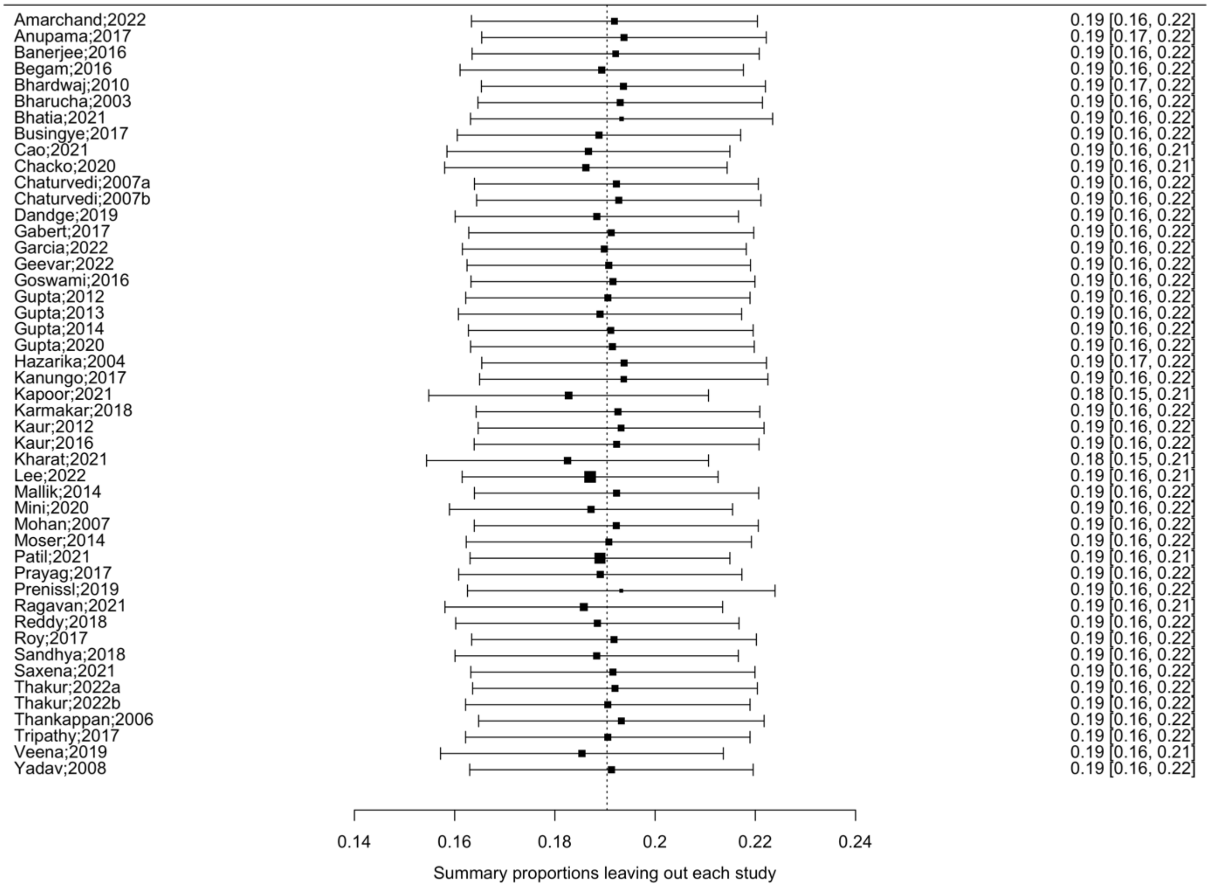

S7 Risk of bias assessment using validated tool by Hoy et al. and summary of level of evidence using SIGN50

Study	Was the study's target population a close representation of the national population?	Was the sampling frame a true or close representation of the target population?	Was random selection used OR, a census used?	Was the likelihood of non-response bias minimal?	Were data collected directly from the subjects?	Was an acceptable case definition used?	Was the study instrument shown to have reliability and validity?	Was the same mode of data collection used for all subjects?	Was the length of the shortest prevalence period appropriate?	Were the numerator and denominator appropriate?	Summary item on the overall risk of study bias?[1]	SIGN 50 score[2]
Amarchand;2022	Low	Low	Low	Low	Low	Low	Low	Low	Low	Low	Low	LE2++
Anupama;2017	High	Low	Low	Low	Low	Low	Low	Low	Low	Low	Low	LE2++
Banerjee;2016	High	Low	Low	Low	Low	Low	Low	Low	Low	Low	Low	LE2++
Begam;2016	High	Low	Low	Low	Low	Low	Low	Low	Low	Low	Low	LE2++
Bhardwaj;2010	High	Low	Low	Low	Low	Low	Low	Low	Low	Low	Low	LE2++
Bharucha;2003	High	Low	Low	Low	Low	Low	Low	Low	Low	Low	Low	LE2++
Bhatia;2021	Low	Low	Low	High	Low	Low	Low	Low	Low	Low	Low	LE2++
Busingye;2017	High	Low	Low	High	Low	Low	Low	Low	Low	Low	Moderate	LE2++
Cao;2021	High	Low	Low	Low	Low	Low	Low	Low	Low	Low	Low	LE2++
Chacko;2020	High	Low	Low	Low	Low	Low	Low	Low	Low	Low	Low	LE2++
Chaturvedi;2007	High	Low	Low	High	Low	Low	Low	Low	Low	Low	Moderate	LE2++
Chaturvedi;2007	High	Low	Low	High	Low	Low	Low	Low	Low	Low	Moderate	LE2++
Dandge;2019	High	Low	Low	High	Low	Low	Low	Low	Low	Low	Moderate	LE2++
Gabert;2017	Low	Low	Low	High	Low	Low	Low	Low	Low	Low	Low	LE2++
Garcia;2022	Low	High	High	High	Low	Low	Low	Low	Low	Low	Moderate	LE2
Geevar;2022	High	Low	Low	Low	Low	Low	Low	Low	Low	Low	Low	LE2++
Godara;2021	High	Low	Low	High	Low	Low	Low	Low	Low	Low	Moderate	LE2++
Goswami;2016	High	Low	Low	Low	Low	Low	Low	Low	Low	Low	Low	LE2++
Gupta;2012	Low	Low	Low	High	Low	Low	Low	Low	Low	Low	Low	LE2++
Gupta;2013	Low	Low	Low	Low	Low	Low	Low	Low	Low	Low	Low	LE2++

Study	Was the study's target population a close representation of the national population?	Was the sampling frame a true or close representation of the target population?	Was random selection used OR, a census used?	Was the likelihood of non-response bias minimal?	Were data collected directly from the subjects?	Was an acceptable case definition used?	Was the study instrument shown to have reliability and validity?	Was the same mode of data collection used for all subjects?	Was the length of the shortest prevalence period appropriate?	Were the numerator and denominator appropriate?	Summary item on the overall risk of study bias?[1]	SIGN 50 score[2]
Gupta;2015	Low	Low	Low	Low	Low	Low	Low	Low	Low	Low	Low	LE2++
Gupta;2020	High	Low	Low	Low	Low	Low	Low	Low	Low	Low	Low	LE2++
Hazarika;2004	High	Low	Low	Low	Low	Low	Low	Low	Low	Low	Low	LE2++
Kanungo;2017	High	Low	Low	High	Low	Low	Low	Low	Low	Low	Moderate	LE2++
Kapoor;2021	High	High	High	Low	Low	Low	Low	Low	Low	Low	High	LE2
Karmakar;2018	High	Low	Low	High	Low	Low	Low	Low	Low	Low	Moderate	LE2++
Kaur;2012	High	Low	Low	Low	Low	Low	Low	Low	Low	Low	Low	LE2++
Kaur;2016	High	Low	Low	Low	Low	Low	Low	Low	Low	Low	Low	LE2++
Kharat;2021	High	Low	Low	Low	Low	Low	Low	Low	Low	Low	Low	LE2++
Kusuma;2013	High	Low	Low	High	Low	Low	Low	Low	Low	Low	Moderate	LE2++
Lee;2022	Low	Low	Low	Low	Low	Low	Low	Low	Low	Low	Low	LE2++
Mallik;2014	High	Low	Low	Low	Low	Low	Low	Low	Low	Low	Low	LE2++
Mini;2020	High	Low	Low	High	Low	Low	Low	Low	Low	Low	Moderate	LE2++
Misra;2014	High	Low	Low	Low	Low	Low	Low	Low	Low	Low	Low	LE2++
Mohan;2007	High	Low	Low	Low	Low	Low	Low	Low	Low	Low	Low	LE2++
Moser;2014	High	Low	Low	Low	Low	Low	Low	Low	Low	Low	Low	LE2++
Patil;2021	Low	Low	Low	Low	Low	Low	Low	Low	Low	Low	Low	LE2++
Prayag;2017	High	Low	Low	Low	Low	Low	Low	Low	Low	Low	Low	LE2++
Prenissl;2019	Low	Low	Low	High	Low	Low	Low	Low	Low	Low	Low	LE2++
Ragavan;2021	High	Low	Low	High	Low	Low	Low	Low	Low	Low	Moderate	LE2++
Reddy;2018	High	Low	Low	High	Low	Low	Low	Low	Low	Low	Moderate	LE2++
Roy;2017	High	Low	Low	Low	Low	Low	Low	Low	Low	Low	Low	LE2++
Sandhya;2018	High	Low	Low	High	Low	Low	Low	Low	Low	Low	Moderate	LE2++

Study	Was the study's target population a close representation of the national population?	Was the sampling frame a true or close representation of the target population?	Was random selection used OR, a census used?	Was the likelihood of non-response bias minimal?	Were data collected directly from the subjects?	Was an acceptable case definition used?	Was the study instrument shown to have reliability and validity?	Was the same mode of data collection used for all subjects?	Was the length of the shortest prevalence period appropriate?	Were the numerator and denominator appropriate?	Summary item on the overall risk of study bias?[1]	SIGN 50 score[2]
Sathish;2012	High	Low	Low	Low	Low	Low	Low	Low	Low	Low	Low	LE2++
Saxena;2021	High	Low	Low	Low	Low	Low	Low	Low	Low	Low	Low	LE2++
Thakur;2022a	High	Low	Low	Low	Low	Low	Low	Low	Low	Low	Low	LE2++
Thakur;2022b	High	Low	Low	Low	Low	Low	Low	Low	Low	Low	Low	LE2++
Thankappan;200[illegible]	High	Low	Low	Low	Low	Low	Low	Low	Low	Low	Low	LE2++
Tripathy;2017	High	Low	Low	Low	Low	Low	Low	Low	Low	Low	Low	LE2++
Veena;2019	High	Low	Low	High	Low	Low	Low	Low	Low	Low	Moderate	LE2++
Yadav;2008	High	Low	Low	Low	Low	Low	Low	Low	Low	Low	Low	LE2++

[1]Hoy et al**:** Hoy D, Brooks P, Woolf A, et al. Assessing risk of bias in prevalence studies: modification of an existing tool and evidence of interrater agreement. *Journal of Clinical Epidemiology*. 2012;65(9):934-939. doi:10.1016/j.jclinepi.2011.11.014
[2]de Winter CF, Jansen AAC, Evenhuis HM. Physical conditions and challenging behaviour in people with intellectual disability: a systematic review. *Journal of Intellectual Disability Research*. 2011;55(7):675-698. doi:10.1111/j.1365-2788.2011.01390.x

S8 *Forest plots showing sub-group analysis across region, study period and sex.*

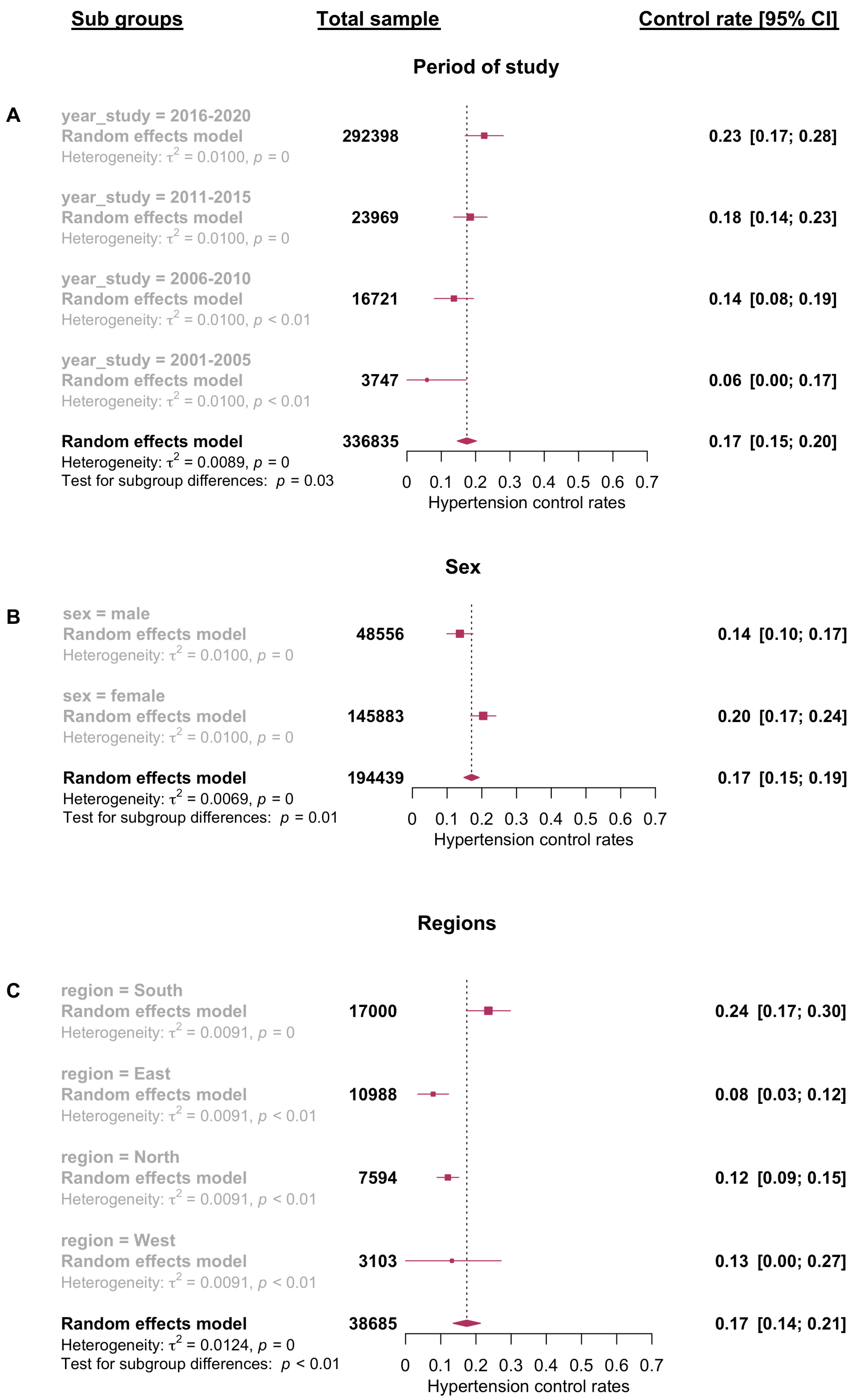

S9 Hypertension control rates in India, 2001-2020: meta-regression model

	**Effect estimate** *(difference in control rate)*	**95% CI**	**p-value**
Intercept	2.9	-4.5,10.4	0.43
Sex (Ref: Female)			
Male	-7.3	-11.5,-3.1	**<0.001**
Region (Ref: East)			
North	2.7	-4.8,10.2	0.47
South	14.2	7.2,21.1	**<.0001**
West	4.8	-3.4,13.1	0.25
Time period (Ref: 2001-2005)			
2006-2010	2.7	-5.6,10.9	0.52
2011-2015	13.4	6.7,20.1	**<0.0001**
2016-2020	14.7	6.9,22.5	**<0.001**

CI: Confidence interval

S10—A

Result of sensitivity analysis: after excluding studies with data only on elderly

Number of studies combined: k = 39
Number of observations: o = 333747
Number of events: e = 53384

	proportion	95%-CI
Common effect model	0.1209	[0.1199; 0.1220]
Random effects model	0.1757	[0.1401; 0.2114]
Prediction interval		[0.0000; 0.3706]

Quantifying heterogeneity: tau^2 = 0.0090 [0.0049; 0.0197]; tau = 0.0949 [0.0701; 0.1403]
I^2 = 99.8%; H = 22.89

Test of heterogeneity:	Q	d.f.	p-value
	19917.56	38	0

S10—B

Result of sensitivity analysis: with only studies with low risk of bias *(Hoy et al)*

Number of studies combined: k = 33
Number of observations: o = 324335
Number of events: e = 51379

	proportion	95%-CI
Common effect model	0.1219	[0.1208; 0.1230]
Random effects model	0.1534	[0.1224; 0.1845]
Prediction interval		[0.0000; 0.3476]

Quantifying heterogeneity:
tau^2 = 0.0088 [0.0037; 0.0163]; tau = 0.0937 [0.0609; 0.1277]
I^2 = 99.8%; H = 23.65

Test of heterogeneity:

Q	d.f.	p-value
17903.02	32	0
