# Supplementary material for: Hypertension control rate in India: systematic review and meta-analysis of population-level non-interventional studies, 2001–2022
Source: Lancet Reg Health Southeast Asia. 2022 Nov 23;9:100113. doi: 10.1016/j.lansea.2022.100113 (PMC10305851; doi:10.1016/j.lansea.2022.100113)
Supplement: Supplementary File S2 [file mmc2.docx]

**Hypertension control rate in India: Systematic review and meta-analysis of population level non-interventional studies, 2001-2022**

**Shaffi Fazaludeen Koya, DrPH, Zarin Pilakkadavath, DNB, Praseeda Chandran MD et al.**

**Caption for supplementary material:**

S1 Search strategy

S2—A Studies included in the systematic review (n= 51): Key variables extracted

S2—B Map showing the distribution of studies on hypertension control rate, 2001-2022

S2—C Blood pressure measurement details from studies included in the systematic review (n= 51)

S3 Criteria used for SIGN50 score- level of evidence

S4 Forest plot of the initial pooled analysis with 47 studies.

S5—A Diagnostic Bajaut plot

S5—B Test of residual

S6 Forest plot of leave-one-out analysis

S7 Risk of bias assessment using validated tool by Hoy et al. and summary of level of evidence using SIGN50

S8 *Forest plots showing sub-group analysis across region, study period and sex.*

S9 Hypertension control rates in India, 2001-2020: meta-regression model

S10—A Result of sensitivity analysis: after excluding studies with data only on elderly

S10—B Result of sensitivity analysis: with only studies with low risk of bias *(Hoy et al)*
