# Supplementary material for: Hypertension control rate in India: systematic review and meta-analysis of population-level non-interventional studies, 2001–2022
Source: Lancet Reg Health Southeast Asia. 2022 Nov 23;9:100113. doi: 10.1016/j.lansea.2022.100113 (PMC10305851; doi:10.1016/j.lansea.2022.100113)
Supplement: Supplementary File S3 [file mmc3.docx]

**भारत में उच्च रक्तचाप नियंत्रण दर: जनसंख्या स्तर के गैर- हस्तक्षेप अध्ययनों की व्यवस्थित समीक्षा और मेटा-विश्लेषण, 2001-2022**

सार

पृष्ठभूमि: भारत में मृत्यु दर में उच्च रक्तचाप का महत्वपूर्ण योगदान है। जनसंख्या स्तर पर बेहतर उच्च रक्तचाप नियंत्रण दर हासिल करना हृदय रुग्णता और मृत्यु दर को कम करने में महत्वपूर्ण है।

तरीके: हमने पिछले 20 वर्षों में प्रकाशित समुदाय-आधारित, गैर- हस्तक्षेप अध्ययनों की एक व्यवस्थित समीक्षा और मेटा-विश्लेषण किया। हमने PubMed, Embase, और Web of Science डेटाबेस, और ग्रे लिटरेचर की खोज की, और एक सामान्य ढांचे का उपयोग करके डेटा निकाला, और अध्ययन विशेषताओं को संक्षेप में प्रस्तुत किया। हमने अनियंत्रित उच्च रक्तचाप नियंत्रण दरों का उपयोग करके यादृच्छिक-प्रभाव मेटा-विश्लेषण किया और समग्र सारांश अनुमानों और नियंत्रण दरों के उपसमूह अनुमानों को प्रतिशत और 95% विश्वास अंतराल के रूप में रिपोर्ट किया। हमने सहसंयोजकों के रूप में सेक्स, क्षेत्र और अध्ययन अवधि के साथ मिश्रित-प्रभाव वाले मेटा-रिग्रेशन का भी संचालन किया। पूर्वाग्रह के जोखिम का आकलन किया गया था, और साक्ष्य के स्तर को SIGN-50 पद्धति का उपयोग करके संक्षेप में प्रस्तुत किया गया था। प्रोटोकॉल PROSPERO, CRD42021267973 के साथ पंजीकृत किया गया था।

निष्कर्ष: व्यवस्थित समीक्षा में 51 अध्ययन (एन = 338,313 उच्च रक्तचाप वाले रोगी) शामिल थे। 21 अध्ययनों (41%) ने महिलाओं की तुलना में पुरुषों के बीच खराब नियंत्रण दर की सूचना दी, और छह अध्ययनों (12%) ने ग्रामीण रोगियों में खराब नियंत्रण दर की सूचना दी। बहुत कम अध्ययनों ने सामाजिक आर्थिक चर या जीवन शैली जोखिम कारकों पर डेटा की सूचना दी। समग्र नियंत्रण दर पर्याप्त विविधता (I^2^=99.8%) के साथ 17.5% (95% विश्वास अंतराल: 14.3% - 20.6%) थी। उप-समूह विश्लेषण ने राष्ट्रीय कार्यक्रम की शुरुआत के बाद नियंत्रण दरों में उल्लेखनीय वृद्धि, दक्षिण और पश्चिम क्षेत्रों में काफी बेहतर नियंत्रण दर और पुरुषों के बीच काफी कम दरों को दिखाया।

व्याख्या: भारत में उच्च रक्तचाप से ग्रस्त रोगियों में से एक-पांचवें से भी कम का रक्तचाप नियंत्रण में था। हालांकि हाल के वर्षों में नियंत्रण दर में सुधार हुआ है, लेकिन क्षेत्रों में पर्याप्त अंतर मौजूद है। बहुत कम अध्ययनों ने भारत में उच्च रक्तचाप नियंत्रण के लिए प्रासंगिक जीवनशैली जोखिम कारकों और सामाजिक निर्धारकों की जांच की है। उच्च रक्तचाप नियंत्रण दरों में सुधार के लिए देश को समुदाय-आधारित रणनीतियों और कार्यक्रमों को विकसित और परीक्षण करने की आवश्यकता है।
